# Incidence and Survival of Multiple Primary Cancers in US Women With a Gynecologic Cancer

**DOI:** 10.3389/fonc.2022.842441

**Published:** 2022-03-23

**Authors:** Logan Corey, Julie Ruterbusch, Ron Shore, Martins Ayoola-Adeola, Michael Baracy, Alex Vezina, Ira Winer

**Affiliations:** ^1^ Department of Oncology, Wayne State University School of Medicine, Detroit, MI, United States; ^2^ Department of OB/GYN, Detroit Medical Center Graduate Medical Education, Detroit, MI, United States; ^3^ Department of Gynecologic Oncology, Karmanos Cancer Institute, Detroit, MI, United States; ^4^ Department of OB/GYN, Ascension St. John Hospital, Detroit, MI, United States; ^5^ Department of OB/GYN, Ochsner Clinic Foundation, New Orleans, LA, United States

**Keywords:** multiple primaries neoplasms gynecological, survivorship (public health), surveillance, gynecologic oncology, women’s cancer

## Abstract

**Objectives:**

To evaluate risk of a second cancer and associated survival times in United States women with diagnosis of cancer.

**Methods:**

The Surveillance Epidemiology and End Results (SEER) database was queried for 2 cohorts of women aged 18 - 89 with either an index gynecologic or non-gynecologic cancer diagnosed between 1992 – 2017. Index cases were followed to determine if a second primary cancer was subsequently diagnosed; defined according to SEER multiple primary and histology coding rules. Standard Incident Ratios (SIR) and latency intervals between index diagnosis and second primary diagnosis were evaluated. Among those who developed a second primary cancer, median survival times from diagnosis of second primary cancer were also calculated.

**Results:**

Between 1992 – 2017, 227,313 US women were diagnosed with an index gynecological cancer and 1,483,016 were diagnosed with an index non-gynecologic cancer. Among patients with index gynecologic cancer, 7.78% developed a non-gynecologic subsequent primary cancer. The risk of developing any non-gynecologic cancer following an index gynecologic cancer was higher than the risk in the general population (SIR 1.05, 95% CI 1.04 - 1.07). Organs especially at risk were Thyroid (SIR 1.45), Colon and Rectum (SIR 1.23), and Urinary System (SIR 1.33). Among women diagnosed with an index non-gynecologic cancer, 0.99% were diagnosed with a subsequent gynecologic cancer. The risk of developing a gynecologic cancer following a non-gynecologic cancer was also elevated compared to the average risk of the general population (SIR 1.05, 1.03 - 1.07), with uterine cancer having the highest SIR of 1.13.

**Conclusion:**

The risk of a developing a second primary cancer and the corresponding survival time is based on the order and site of the index and subsequent cancer. Surveillance guidelines should be examined further to optimize survivorship programs.

## Highlights

Question:

What are the rates of non-gynecologic cancers in survivors of gynecologic cancer, and vice versa?

Findings:

Female cancer survivors are at an increased risk of certain site-specific second primary cancers, and the risk and associated median survival vary based on the order and sites of diagnosis of the index and subsequent primary cancers.

Meaning:

Survivorship is a team effort and all providers who take part should be aware of the specific second primary cancers that cancers are at risk of developing, and the associated survival.

## Introduction

Cancer survival has improved over the last three decades with an estimated 22.2 million cancer survivors by 2030 (1). The cancer mortality rate is higher among women than men, but improved survival has resulted in nearly 1,000,000 female cancer-related deaths averted since 1991 (2). Cancer survivors are at an elevated risk of developing a second primary cancer compared to the general population, although the exact risk varies based on cancer type, etiological exposures, and effects of chemotherapy or radiation (3, 4).

Cancer survivorship is a team effort and all encounters with a patient provide an opportunity to screen for recurrent or new cancers in at-risk populations. Fewer patients are seeing both a general practitioners and specialists, which may shift some general screening responsibilities onto subspecialists (5, 6). Alternatively, long-term cancer survivors may be discharged from their original oncologists to be followed solely by their primary care providers. With an increasing number of multiple primary cancers in the US female population, all providers of women’s healthcare should be aware of the risks of their patients’ developing a second cancer and adjust surveillance accordingly (7).

While there have been a number of studies evaluating the risk of a second cancer in site-specific cancer survivor populations (8–14), few studies focus on second primary cancer incidences and survival rates specific to women (e.g., gynecologic). The aim of this study was to evaluate the risk of developing a second primary gynecologic cancer (GC) following an index diagnosis of a non-gynecologic cancer (NGC), as well as the risk of developing a second primary NGC following the diagnosis of index GC. Additionally, we examined the associated survival for each group.

## Materials and Methods

This is a retrospective cohort study examining the risk of multiple primary cancers in female patients after diagnosis of either GC or NGC index cancer between 1992 - 2017 using the National Cancer Institute’s Surveillance Epidemiology and End Results (SEER) program. SEER is a publicly available data source capturing about 97% of all incident cancers in its registry areas. The SEER 13 dataset was used. For this study, first primary site cancers were coded according to the International Classification of Diseases for Oncology (ICDO). The study cohort included female patients aged 18-89 years diagnosed with index cancer of any site. This study was deemed to be a non-human participant study and exempt for full review by Wayne State University IRB (IRB 2021 – 012).

Two cohorts were evaluated. The first (C1) consisted of female patients aged 18-89 with index cancer from a gynecologic cancer site: cervical (coded as cervix uteri in SEER), endometrial (coded as corpus uteri and uterus, not otherwise specified (NOS) in SEER), ovarian (coded as ovarian, peritoneum, and fallopian tube in SEER) vaginal/vulvar cancers (coded as vulvar or vaginal cancers in SEER), and other female genital organ (coded as broad ligament, round ligament, parametrium, uterine adnexa, other specified parts of female genital organs, overlapping lesions of female genital organs, female genital tract NOS, and placenta). Cases identified by death certificate only were excluded.

The second cohort (C2) consisted of female patients aged 18-89 with index cancer from any site excluding the aforementioned gynecologic sites as well as SEER determined “other female genital organs” cancer. Patients identified by death certificate only were excluded.

We defined a second primary cancer according to the SEER Multiple Primary and Histology Coding rules (15). A second primary cancer was defined as a new primary cancer occurring at least 2 months after an index cancer. Second cancers that developed as a result of recurrences, extensions, or metastasis were excluded. For C1 we only included second cancers from non-gynecologic sites. We performed a reciprocal analysis on C2 and only included second cancers arising from gynecologic sites.

### Statistical Analysis

A case-listing session in SEER*Stat was used to identify each of the cohorts and descriptive and survival analyses were completed using SAS version 9.4 software (Cary, NC). In addition, the R package “cmprsk” (16) was used to draw cumulative incidence graphs for the development of a second primary cancer. Survival time was calculated from diagnoses to death or last contact, and median survival was calculated using the Kaplan-Meier method. Median survival was calculated overall for each cohort and stratified by the most common cancer sites for both the index and outcome cancer sites. In addition, median survival was calculated by years of subsequent cancer diagnosis (1992-1999; 2000-2009; 2010-2018). Standardized incidence ratio (SIR) analyses were conducted using SEER*Stat software. SIRs were calculated separately for each cohort by comparing the observed occurrence of second primary cancer to the expected number of cancers based on incidence rates in the general population of the respective SEER areas. SIRs were calculated overall, and stratified by latency period (<5 years, ≥5 years), and for the most common cancer sites among each of the subsequent cancer groups.

## Results

### Study Population

Between 1992 and 2017, 227,313 female patients were diagnosed with an index GC and 1,483,016 were diagnosed with an index NGC. Among the patients with index GC, 7.78% developed a non-gynecologic subsequent primary cancer. Among those diagnosed with index NGC, 0.99% were diagnosed with a subsequent GC. Patients diagnosed with index GC presented with local stage more frequently than patients with index NGC (52.8% *vs* 47.4%). Of those who developed a second GC (following index NGC), uterine cancer was the most common. Of those who developed a second NGC (following index GC), breast cancer was the most common.

### Risk of Second Primary Cancers (SPCs)

**Tables 1** and **2** present the risks of developing a subsequent cancer using standardized incidence ratios (SIR). The rates of developing SPCs are evaluated overall and are also stratified by latency interval (<5 years and >=5 years). **Table 3** demonstrates rates that survivors of GC develop a second primary NGC. Overall, the risk of developing any NGC following an index GC is significantly higher than expected when compared to the general population (SIR 1.05, 95% CI 1.04 - 1.07). Interestingly, this risk was present within 5 years of the first primary cancer (SIR 1.13, 95% CI 1.11 - 1.16) and not after five years (SIR 0.99, 95% CI 0.97 - 1.01).

**Table 1:** Index GYN cancer and risk of subsequent non-GYN cancer.

Index Site	All Non GYN Sites		Breast		Lung and Bronchus		Colon and Rectum		Thyroid		Hematopoietics		Urinary System	
	SIR	95% CI	SIR	95% CI	SIR	95% CI	SIR	95% CI	SIR	95% CI	SIR	95% CI	SIR	95% CI
Total														
All GYN Sites	**1.05**	(1.04, 1.07)	0.98	(0.95, 1.00)	0.97	(0.93, 1.00)	**1.23**	(1.18, 1.28)	**1.45**	(1.34, 1.56)	1.03	(0.98, 1.08)	**1.33**	(1.26, 1.41)
Cervix Uteri	**1.19**	(1.14, 1.23)	0.75	(0.69, 0.81)	**2.10**	(1.93, 2.28)	**1.30**	(1.15, 1.46)	**1.21**	(1.00, 1.46)	1.10	(0.95, 1.26)	**1.76**	(1.52, 2.03)
Corpus and Uterus, NOS	**1.02**	(1.00, 1.03)	1.03	(0.99, 1.06)	0.77	(0.73, 0.81)	**1.28**	(1.22, 1.35)	**1.47**	(1.33, 1.63)	0.99	(0.93, 1.05)	**1.28**	(1.19, 1.37)
Ovary	**1.03**	(1.00, 1.07)	0.98	(0.92, 1.04)	0.77	(0.69, 0.86)	1.08	(0.97, 1.21)	**1.58**	(1.31, 1.88)	**1.17**	(1.04, 1.31)	**1.24**	(1.07, 1.42)
Vulva/Vaginal	**1.27**	(1.20, 1.35)	0.98	(0.87, 1.11)	**1.95**	(1.71, 2.2)	1.00	(0.82, 1.21)	**1.89**	(1.3, 2.66)	0.99	(0.78, 1.23)	**1.54**	(1.22, 1.93)
2-59 months														
All GYN Sites	**1.13**	(1.11, 1.16)	1.01	(0.97, 1.05)	**1.09**	(1.02, 1.15)	**1.23**	(1.15, 1.31)	**1.97**	(1.78, 2.18)	1.06	(0.99, 1.15)	**1.64**	(1.52, 1.78)
Cervix Uteri	**1.44**	(1.35, 1.53)	0.79	(0.69, 0.91)	**2.76**	(2.43, 3.13)	**1.27**	(1.03, 1.54)	**1.87**	(1.42, 2.40)	1.22	(0.95, 1.53)	**3.10**	(2.54, 3.74)
Corpus and Uterus, NOS	**1.12**	(1.09, 1.15)	**1.09**	(1.04, 1.15)	0.90	(0.83, 0.98)	**1.33**	(1.23, 1.43)	**1.98**	(1.73, 2.26)	1.06	(0.96, 1.16)	**1.54**	(1.39, 1.70)
Ovary	0.99	(0.94, 1.04)	0.86	(0.78, 0.95)	0.74	(0.63, 0.86)	1.02	(0.87, 1.18)	**1.90**	(1.49, 2.39)	1.04	(0.87, 1.23)	**1.34**	(1.10, 1.63)
Vulva/Vaginal	**1.35**	(1.24, 1.47)	1.07	(0.90, 1.27)	**1.97**	(1.64, 2.35)	0.88	(0.65, 1.17)	**2.62**	(1.62, 4.00)	0.90	(0.63, 1.25)	**1.93**	(1.41, 2.57)
60+ months														
All GYN Sites	0.99	(0.97, 1.01)	0.95	(0.92, 0.98)	0.88	(0.83, 0.93)	**1.24**	(1.17, 1.31)	1.05	(0.92, 1.18)	1.01	(0.94, 1.07)	**1.12**	(1.03, 1.21)
Cervix Uteri	**1.06**	(1.00, 1.11)	0.73	(0.66, 0.81)	**1.77**	(1.58, 1.97)	**1.32**	(1.13, 1.52)	0.86	(0.65, 1.13)	1.04	(0.86, 1.23)	1.13	(0.90, 1.40)
Corpus and Uterus, NOS	0.95	(0.92, 0.97)	0.98	(0.93, 1.02)	0.68	(0.63, 0.73)	**1.25**	(1.17, 1.34)	1.07	(0.90, 1.25)	0.94	(0.87, 1.02)	**1.11**	(1.01, 1.22)
Ovary	**1.08**	(1.03, 1.14)	**1.10**	(1.01, 1.20)	0.80	(0.68, 0.94)	1.16	(0.99, 1.35)	1.26	(0.94, 1.67)	**1.30**	(1.11, 1.51)	1.13	(0.91, 1.39)
Vulva/Vaginal	**1.20**	(1.09, 1.31)	0.90	(0.74, 1.08)	**1.93**	(1.61, 2.29)	1.13	(0.86, 1.46)	1.28	(0.66, 2.23)	1.07	(0.78, 1.43)	1.19	(0.81, 1.69)

Bolded: Lower end of 95% CI >=1.

**Table 2:** Index non-GYN cancer and risk of subsequent GYN cancer.

Index Site	All GYN Sites		Cervix Uteri		Corpus and Uterus, NOS		Ovary		Vulva/Vaginal	
	SIR	95% CI	SIR	95% CI	SIR	95% CI	SIR	95% CI	SIR	95% CI
Total										
All Non GYN Sites	**1.05**	(1.03, 1.07)	0.71	(0.66, 0.75)	**1.13**	(1.11, 1.15)	1.02	(0.98, 1.05)	0.96	(0.91, 1.02)
Breast	**1.13**	(1.11, 1.16)	0.57	(0.51, 0.63)	**1.25**	(1.22, 1.29)	**1.12**	(1.08, 1.17)	0.76	(0.68, 0.83)
Lung and Bronchus	0.65	(0.59, 0.72)	0.62	(0.43, 0.88)	0.50	(0.43, 0.58)	0.91	(0.77, 1.06)	0.84	(0.61, 1.13)
Colon and Rectum	**1.13**	(1.08, 1.18)	1.10	(0.93, 1.3)	**1.33**	(1.26, 1.41)	0.80	(0.72, 0.89)	1.04	(0.88, 1.23)
Thyroid	1.01	(0.93, 1.09)	0.64	(0.48, 0.84)	1.09	(0.98, 1.2)	1.08	(0.92, 1.27)	0.82	(0.55, 1.17)
Hematopoietics	0.92	(0.86, 0.98)	0.90	(0.72, 1.11)	0.90	(0.83, 0.98)	0.92	(0.82, 1.04)	1.05	(0.84, 1.29)
Urinary System	1.02	(0.95, 1.09)	1.02	(0.78, 1.32)	1.07	(0.97, 1.17)	0.80	(0.68, 0.94)	**1.28**	(1.02, 1.6)
2-59 months										
All Non GYN Sites	**1.06**	(1.03, 1.09)	0.82	(0.75, 0.90)	**1.10**	(1.06, 1.14)	**1.05**	(1.00, 1.10)	1.03	(0.94, 1.13)
Breast	**1.12**	(1.08, 1.16)	0.64	(0.55, 0.74)	**1.22**	(1.16, 1.27)	**1.16**	(1.09, 1.24)	0.72	(0.61, 0.85)
Lung and Bronchus	0.62	(0.55, 0.70)	0.65	(0.41, 0.97)	0.46	(0.38, 0.55)	0.89	(0.72, 1.08)	0.82	(0.54, 1.20)
Colon and Rectum	**1.17**	(1.10, 1.25)	**1.34**	(1.08, 1.64)	**1.33**	(1.22, 1.45)	0.83	(0.72, 0.97)	1.09	(0.85, 1.38)
Thyroid	1.11	(0.98, 1.26)	0.71	(0.46, 1.05)	**1.23**	(1.04, 1.43)	1.19	(0.92, 1.51)	1.02	(0.54, 1.74)
Hematopoietics	0.92	(0.84, 1.00)	0.90	(0.66, 1.20)	0.87	(0.77, 0.98)	1.01	(0.86, 1.19)	0.92	(0.65, 1.27)
Urinary System	**1.14**	(1.04, 1.26)	1.25	(0.88, 1.72)	**1.18**	(1.04, 1.35)	0.92	(0.73, 1.13)	**1.60**	(1.16, 2.14)
60+ months										
All Non GYN Sites	**1.05**	(1.02, 1.07)	0.59	(0.53, 0.66)	**1.15**	(1.12, 1.19)	0.98	(0.94, 1.03)	0.92	(0.84, 0.99)
Breast	**1.14**	(1.10, 1.17)	0.51	(0.43, 0.59)	**1.28**	(1.23, 1.32)	**1.10**	(1.04, 1.16)	0.77	(0.68, 0.87)
Lung and Bronchus	0.70	(0.60, 0.82)	0.56	(0.26, 1.07)	0.58	(0.46, 0.73)	0.95	(0.72, 1.23)	0.87	(0.51, 1.40)
Colon and Rectum	**1.09**	(1.02, 1.16)	0.83	(0.62, 1.09)	**1.33**	(1.23, 1.44)	0.76	(0.66, 0.89)	1.01	(0.80, 1.26)
Thyroid	0.94	(0.85, 1.04)	0.58	(0.38, 0.85)	1.01	(0.89, 1.15)	1.02	(0.82, 1.25)	0.71	(0.42, 1.14)
Hematopoietics	0.92	(0.84, 1.00)	0.90	(0.65, 1.21)	0.94	(0.83, 1.05)	0.83	(0.69, 1.00)	1.16	(0.87, 1.53)
Urinary System	0.90	(0.81, 1.00)	0.78	(0.48, 1.19)	0.96	(0.83, 1.10)	0.69	(0.54, 0.88)	1.03	(0.72, 1.43)

Bolded: Lower end of 95% CI >=1.

**Table 3:** Median time in months to development of subsequent cancer.

Index Cancer = Gyn	
Subsequent Cancer	
All Non GYN Sites	68
Breast	67
Lung and Bronchus	65
Colon and Rectum	72
Thyroid	40
Hematopoietics	74
Urinary System	59
**Index Cancer = non-Gyn**	
Subsequent Cancer	
All GYN Sites	68
Cervix Uteri	44
Corpus and Uterus, NOS	71
Ovary (including Fallopian Tube)	64
Vulva/Vaginal	70

Of all index GCs, survivors of cervical and vulva or vaginal cancer had the highest risk of developing a second primary NGC (SIR 1.19, 95% CI 1.14 – 1.23 and 1.27, 95% CI 1.20 – 1.35, respectively). These patients were at significantly higher risk than the average population of developing second lung and bronchus cancers (SIR_(cervix)_ 2.10, SIR_(vulva/vagina)_ 1.95), thyroid cancer (SIR_(cervix)_ 1.21, SIR_(vulva/vagina)_ 1.89), and cancers of the urinary system (SIR_(cervix)_ 1.76, SIR_(vulva/vagina)_ 1.54). When stratified by individual index GC type, each type of GC carried a significantly increased risk of developing cancers of the urinary system and thyroid compared to the general population.

The risk of developing GC following an index NGC was significantly elevated compared to the general population (SIR 1.05, 95% CI 1.03 - 1.07). Survivors with an index cancer of the breast (SIR 1.13, 95% CI 1.11 - 1.16) and colon and rectum (SIR 1.13, 95% CI 1.08 - 1.18) were at highest risk of developing a second primary GC. The risk of developing cervical cancer was significantly lower in survivors of NGCs compared to the general population (SIR 0.71, 95% CI 0.66-0.75). The risk of developing a subsequent uterine cancer was highest in patients with index cancers of the breast (SIR 1.25, 95% CI 1.22 - 1.29) and colon and rectum (SIR 1.33, 95% CI 1.26 - 1.41). There was no increased risk of ovarian or vulvar/vaginal cancer following diagnosis of NGC index primary, except in patients diagnosed with a cancer of the urinary system. These patients had significantly increased risk only in developing cancers of the vulva or vagina (SIR 1.28, 95% CI 1.02 - 1.60).

### Latency Intervals

Latency period (the time between cancer diagnoses) was also found to have an impact on SIR. Patients with index uterine cancer had a significantly higher risk of developing a second NGC between 2-59 months after the index cancer diagnosis (SIR 1.12, 95% CI 1.09 - 1.15) but this rate fell to below the norm after 60 months (SIR 0.95, 95% CI 0.92 - 0.97). Conversely, survivors of ovarian cancer only had significantly increased risk of a second NGC 60 months or more after index diagnosis (SIR 1.08, 95% CI 1.03 - 1.14), mostly influenced by the increased incidences of hematopoietic cancers in ovarian cancer survivors (SIR 1.30, 95% CI 1.11 - 1.51). Patients with index cervical, ovarian and vulvar or vaginal cancers remained at significantly higher risk of developing any second NGC even beyond 5 years (**Table 3**).

**Table 2** demonstrates patients with an index cancer of the breast or colon and rectum maintained an elevated risk of a subsequent GC 2-59 months after diagnosis (SIR_(breast)_ 1.12, SIR_(colon and rectum)_ 1.17) as well as 60 months or longer following index diagnosis (SIR_(breast)_ 1.14, SIR_(colon and rectum)_ 1.09). The risks of developing a GC after index cancer of the thyroid and urinary system were only elevated in the first five years after index diagnosis (SIR_(thyroid)_ 1.11), SIR_(urinary system)_ 1.14). The risk of developing a subsequent cervical cancer was only elevated in patients within five years of an index diagnosis of colon and rectal cancer (SIR 1.34, 95% CI 1.08 - 1.64). Patients with an index cancer of the urinary system had the highest risk of a second primary vulvar or vaginal cancer within the first five years of diagnosis (SIR 1.60, 95% CI 1.16 - 2.14).

Next, we evaluated the median time to development of a SPC after the index cancer (**Table 3**). The patients with index GC who developed a subsequent NGC had a median latency period of 68 months and a range of 40 months (thyroid cancer) to 74 months (hematopoietic cancers). The patients with index NGC who developed a subsequent GC had a median latency of 68 months and a range of 44 months (cervical cancer) to 70 months (vulvar/vaginal cancer).

**Figure 1:**
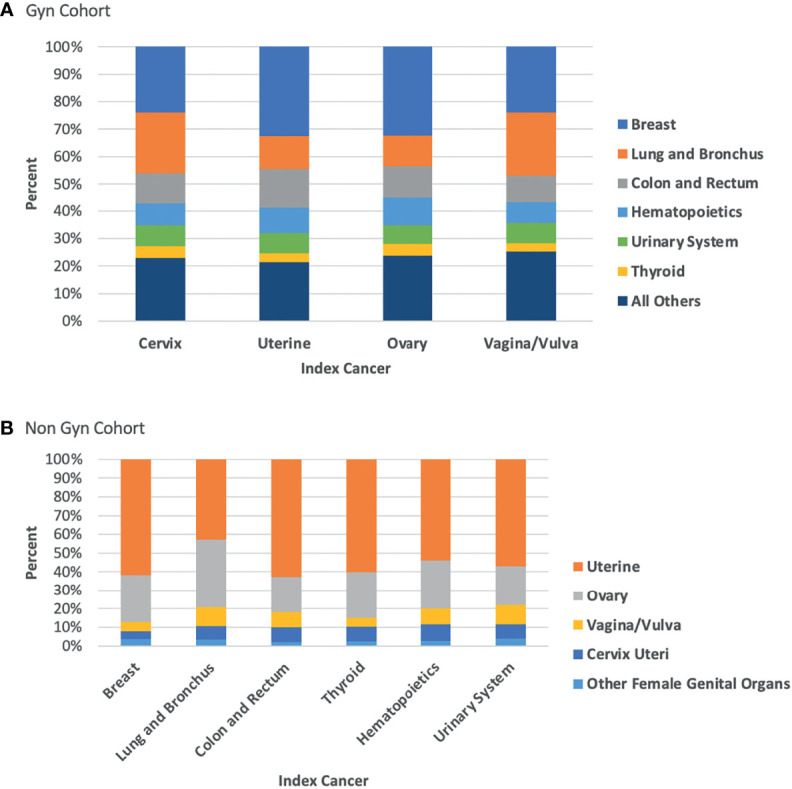
**(A)** Proportion of second primary cancer site by index GC; **(B)** Proportion of second primary cancer site by index NGC.

**Figures 1A, B** demonstrate the proportional makeup of second primary cancers separated by index cancer type. Interestingly, in the population of patients with an index NGC, only cancers of the lung and bronchus did not have over 50% of the proportion of subsequent second primary cancer attributed to endometrial, accounting for only 43%. This observation can be attributed to the increased proportion of subsequent ovarian cancer in this group (37%) compared to the proportion in other types of index NGC (19-26%).

### Survival

**Table 4** depicts the median survival from the subsequent SPC diagnosis. Patients with an index GC had a median survival of 60 months following diagnosis of a subsequent NGC ranging from 12 months (cancer of the lung and bronchus) to 147 months (breast cancer). Patients with vaginal or vulvar cancer and an SPC of the lung and bronchus had the shortest median survival from the subsequent diagnosis (9 months). Interestingly, patients with vaginal or vulvar cancer and a second primary thyroid cancer had the longest median survival from the subsequent diagnosis (240 months). In general, survival from a second primary thyroid cancer diagnosis following any GC was too long to be calculated (i.e., the median time could not be calculated because half of the cohort was still alive at time of analysis). Survival from a SPC diagnosis of the lung and bronchus following any index GC was lowest and ranged between 9-14 months. Patients diagnosed with a subsequent urinary system cancer had the widest relative range depending on the index GC type, ranging from 39 months (cervical cancer) to >2x longer median survival of 85 months in patients with index endometrial cancer.

**Table 4:** Median Survival in months from Subsequent Cancer Diagnosis by Index Cancer.

Index Cancer = Gyn	All GYN Sites	Cervix Uteri	Corpus and Uterus, NOS	Ovary	Vulva/Vaginal		
***Subsequent Cancer***							
All Non GYN Sites	60	42	73	40	37		
Breast	147	199	152	113	122		
Lung and Bronchus	12	10	14	12	9		
Colon and Rectum	63	58	70	41	52		
Thyroid	*	*	*	181	240		
Hematopoietics	37	52	41	20	32		
Urinary System	69	39	85	46	45		
**Index Cancer = non-Gyn**	**All Non GYN Sites**	**Breast**	**Lung and Bronchus**	**Colon and Rectum**	**Thyroid**	**Hematopoietics**	**Urinary System**
***Subsequent Cancer***							
All GYN Sites	67	75	25	51	186	45	63
Cervix Uteri	40	48	13	27	*	63	41
Corpus and Uterus, NOS	108	115	55	85	209	81	119
Ovary	30	36	13	21	84	14	20
Vulva/Vaginal	50	56	38	52	215	45	43

*Median survival was not reached and not able to be calculated.

Patients with an index NGC and a subsequent GC had a median survival of 67 months ranging from 30 months (ovary) to 108 months (endometrial cancer). Patients with an index lung and bronchus cancer had shortest median survival after development of ovary or cervical cancer (13 months each) and longest survival after development of endometrial cancer (55 months). Patients with an index thyroid cancer had the longest median survival of any patient group after development of second primary GC (84 - 209 months). Patients diagnosed with a subsequent ovary cancer had the widest relative survival range depending on the index NGC type, ranging from 13 months (cancer of the lung and bronchus) to 84 months in patients with index thyroid cancer (6.46 times longer than lung and bronchus cancers).

The median survival in months from subsequent cancer diagnosis stratified into three strata, based on year of index cancer diagnosis is shown in **Supplementary Table 3**. Nearly all median survival times have increased over the last three decades, regardless of index cancer type. Median survival of patients diagnosed with an index GC and a second primary hematopoietic cancer increased from 24 months in stratum 1 to 42 months in stratum 3 (1.75-fold increase). Median survival of patients diagnosed with an index NGC and subsequent.

## Discussion

Our findings are significant for multiple reasons: First, the data shows that women with either an index GC or index NGC are at elevated risk of developing a reciprocal second primary cancer. Second, the risk of being diagnosed with a second primary cancer and the median survival time after the diagnosis are dependent on the anatomic sites of both the index and subsequent cancer. Third, median survival after diagnosis of a second primary cancer has improved over time. Finally, the findings show that the risk of a subsequent cancer is associated with the interval of time that has passed since index diagnosis.

Our findings support previous reports that patients with a history of cancer tend to be at an elevated risk of developing another cancer compared to the general population (3, 4, 17–22). However, almost none have focused on female-specific cancers. Boakye et al. recently published an analysis on risks of developing a second primary cancer after an index gynecologic cancer but failed to evaluate associated survival (14). We expanded on this analysis to include both GC and NGC as the index cancers, as well as survival.

Cancer survival has been overall improving in US women since 1991. This decline in cancer-related mortality is multifactorial and can be attributed to a combination of lead time bias due to earlier detection, efforts to promote smoking cessation, and improvements in treatments (2). A novel finding in this study is that survival of women with multiple primary cancers is longer in the more recent years of diagnosis (in spite of lead time bias), indicating that the improvement in female cancer mortality in general has translated to improvement in patients who develop multiple primary cancers.

Some studies have shown metachronous and synchronous cancer survivorship depends on both the anatomical site of the cancers as well as the temporal relation (e.g., index lung cancer and subsequent breast cancer patients have increased survival than index breast and subsequent lung cancer) (9). Our study found similar anatomical and temporal relationships to survival. For example, patients with index endometrial cancer and subsequent lung cancer have a median survival of 14 months compared to 55 months for those with reciprocal diagnoses. However, these findings may be confounded by the median ages at time of diagnosis for certain cancers. A patient with thyroid cancer is typically diagnosed at an earlier median age than a patient with lung cancer, which may impact survival times (1).

Despite the increased risk of second primary cancers in cancer survivors, there are few screening guidelines for female cancer survivors beyond surveillance for recurrence of the initial primary cancer. This remains a clinical problem as guideline concordant screening is not strictly followed in average risk populations (23), and is challenging even in patients with higher-than-average risk and well-defined surveillance regimens (24–26). This challenge has been attributed to difficulty coordinating surveillance and preventive care across specialties, as well as lack of screening tests for organs at risks, such as thyroid cancer and endometrial cancers (27).

Both general practitioners and specialists are in a position to assist in coordinating preventative services like cancer screening and appropriate specialist referrals (28). A recent study demonstrated as many as 15% patients with more than one primary cancer have a pathogenic germline variant. Ultimately, these patients should undergo genetic testing and counseling (29). Our study provides data to support clinical providers in fulfilling a crucial role in the care of female survivors of any cancer type as subsequent new primary malignant neoplasms may occur in survivors years after treatment when the survivor’s original oncologist may no longer be involved in the survivor’s care (30).

The major strength of our study is the size and quality of the cohort. SEER registry has a high level of completeness as well as intense quality control. Another strength of our methodology is our separation of gynecologic and non-gynecologic cohorts. Because a surgery involving a hysterectomy typically includes removal of the cervix and often includes removal of the ovaries, it is difficult to account for hysterectomy- or oophorectomy-adjusted rates of gynecologic cancer, which may result in an iatrogenically decreased risk where there is actually an average or higher-than-average risk (31).

Before considering implications of this study’s findings, several methodological limitations must be considered: 1) The rate of second primary malignancies could be overestimated if they were truly recurrences or metastases. We believe this is a low risk given the different anatomical sites studied between index and second cancer, and that the sites of increased risk for a second cancer are not common sites for metastases (e.g., endometrial cancer rarely metastasizes to the breast). 2) SEER*Stat data is limited to possible etiologic linkages of metachronous cancer including BMI, smoking status, or HPV status. 3) Our analyses did not control for treatment regimens – a significant limitation given exposure to some treatments (e.g., radiation) may paradoxically both be an etiology for the next cancer and hamper optimal treatment of the next cancer. 4) Although SEER generalizability is likely, SEER areas have lower percentages of whites and higher percentages of young people, are more urban, and have higher percentage of poor, lower educational attainment, and higher unemployment than non-SEER areas which may limit external validity (32). Lastly, 5) patients with cancers with high mortality rates and who do not live long enough to develop a second primary cancer will have an artificial “protective” effect of their index cancer. This can be seen in patients with index lung cancer in our cohort that we found to be associated with lower rates of subsequent gynecologic cancers, most likely because of a higher mortality rate and the decreased survival interval of the index cancer.

## Conclusion

Our study has major implications for female cancer survivors, whom we have demonstrated are at risk for subsequent cancers at seemingly unrelated anatomical sites. Even as survivorship guidelines for site-specific cancers become more comprehensive, it is important that the providers of female healthcare be aware that these patients are at a higher-than-average risk for a second cancer. With this knowledge, providers will be able to adjust symptom assessment, physical exam, and genetic testing accordingly. We have shown that risk of a second cancer and the corresponding survival time varies based on the order and site of the index and subsequent cancer, as well as the decade of diagnosis.

## Data Availability Statement

Publicly available datasets were analyzed in this study. This data can be found here: SEER*Stat https://seer.cancer.gov/seerstat/.

## Author Contributions

LC: conceptualization, investigation, methodology development, original draft preparation, and supervision. JR: data analysis, data gathering, and methodology. RS: data analysis, data gathering, and methodology. MA-A: writing, editing, and final draft preparation. MB: writing, editing, and final draft preparation. AV: writing, editing, and final draft preparation. IW: conceptualization, investigation, methodology development, editing, final draft preparation, and supervision. All authors contributed to the article and approved the submitted version.

## Funding

Formal statistical analysis and data curation was funded through grants awarded by the Epidemiology Research Core at Karmanos Cancer Institute as part of their Core Incentive Program.

## Conflict of Interest

The authors declare that the research was conducted in the absence of any commercial or financial relationships that could be construed as a potential conflict of interest.

## Publisher’s Note

All claims expressed in this article are solely those of the authors and do not necessarily represent those of their affiliated organizations, or those of the publisher, the editors and the reviewers. Any product that may be evaluated in this article, or claim that may be made by its manufacturer, is not guaranteed or endorsed by the publisher.
