# Supplementary material for: Incidence and Survival of Multiple Primary Cancers in US Women With a Gynecologic Cancer
Source: Front Oncol. 2022 Mar 23;12:842441. doi: 10.3389/fonc.2022.842441 (PMC8983878; doi:10.3389/fonc.2022.842441)
Supplement: Supplementary file 1 [file Table_1.docx]

| Supplementary Table 1. Demographics for each cancer case cohort | | | | | | | | |  | |  | |  | |
| --- | --- | --- | --- | --- | --- | --- | --- | --- | --- | --- | --- | --- | --- | --- |
| Index GYN cancer |  |  |  | |  | |  | |  | |  | |  | |
| Primary Site | All GYN Sites | Cervix Uteri | | Corpus Uteri and Uterus, NOS | | Ovary | | Vulva/Vaginal | |  | |  | |  |
| Total | 227,313 | 39,029 | | 119,192 | | 53,736 | | 11,133 | |  | |  | |  |
| Age of diagnosis of index |  |  | |  | |  | |  | |  | |  | |  |
| 18-49 | 55,625 | 22,103 | | 17,921 | | 12,745 | | 1,917 | |  | |  | |  |
| 50-59 | 55,479 | 7,276 | | 32,754 | | 12,668 | | 1,879 | |  | |  | |  |
| 60-69 | 56,609 | 5,003 | | 35,891 | | 12,422 | | 2,150 | |  | |  | |  |
| 70+ | 59,600 | 4,647 | | 32,626 | | 15,901 | | 5,187 | |  | |  | |  |
| Year of diagnosis of index cancer |  |  | |  | |  | |  | |  | |  | |  |
| 1992-1999 | 63,193 | 13,514 | | 29,959 | | 15,765 | | 3,178 | |  | |  | |  |
| 2000-2009 | 83,916 | 14,619 | | 43,128 | | 20,721 | | 4,168 | |  | |  | |  |
| 2010-2017 | 80,204 | 10,896 | | 46,105 | | 17,250 | | 3,787 | |  | |  | |  |
| Race/Ethnicity |  |  | |  | |  | |  | |  | |  | |  |
| NHW | 150,518 | 19,161 | | 82,915 | | 37,324 | | 8,267 | |  | |  | |  |
| NHB | 20,237 | 4,756 | | 10,147 | | 3,969 | | 1,020 | |  | |  | |  |
| Hispanic | 30,877 | 9,705 | | 13,042 | | 6,508 | | 1,100 | |  | |  | |  |
| NHA | 23,047 | 4,791 | | 11,778 | | 5,421 | | 607 | |  | |  | |  |
| Other/Unknown | 2,634 | 616 | | 1,310 | | 514 | | 139 | |  | |  | |  |
| SEER Summary Stage at diagnosis of index (a) |  |  | |  | |  | |  | |  | |  | |  |
| Local | 120,124 | 19,876 | | 83,902 | | 9,599 | | 5,990 | |  | |  | |  |
| Regional | 48,215 | 13,115 | | 22,406 | | 9,333 | | 2,725 | |  | |  | |  |
| Distant | 47,798 | 4,156 | | 8,973 | | 32,329 | | 593 | |  | |  | |  |
| Unknown | 11,176 | 1,882 | | 3,911 | | 2,475 | | 1,825 | |  | |  | |  |
| Index NON GYN cancer |  |  | |  | |  | |  | |  | |  | |  |
| Primary Site | All Non GYN Sites | Breast | | Lung and Bronchus | | Colon and Rectum | | Thyroid | | Hematopoietics | | Urinary System | |  |
| Total | 1,483,016 | 573,692 | | 164,492 | | 167,405 | | 71,839 | | 133,742 | | 73,231 | |  |
| Age of diagnosis of index |  |  | |  | |  | |  | |  | |  | |  |
| 18-49 | 315,957 | 141,886 | | 10,523 | | 18,641 | | 41,933 | | 28,497 | | 8,494 | |  |
| 50-59 | 296,268 | 139,169 | | 26,923 | | 28,402 | | 14,794 | | 21,885 | | 12,455 | |  |
| 60-69 | 335,406 | 134,078 | | 46,961 | | 35,705 | | 9,052 | | 28,620 | | 17,880 | |  |
| 70+ | 535,385 | 158,559 | | 80,085 | | 84,657 | | 6,060 | | 54,740 | | 34,402 | |  |
| Year of diagnosis of index cancer |  |  | |  | |  | |  | |  | |  | |  |
| 1992-1999 | 400,572 | 160,116 | | 48,113 | | 52,080 | | 12,098 | | 35,167 | | 18,689 | |  |
| 2000-2009 | 572,425 | 218,133 | | 63,848 | | 66,334 | | 27,585 | | 52,238 | | 28,413 | |  |
| 2010-2017 | 510,019 | 195,443 | | 52,531 | | 48,991 | | 32,156 | | 46,337 | | 26,129 | |  |
| Race/Ethnicity |  |  | |  | |  | |  | |  | |  | |  |
| NHW | 1,031,230 | 397,729 | | 122,692 | | 112,737 | | 44,076 | | 92,201 | | 52,633 | |  |
| NHB | 142,670 | 55,142 | | 17,880 | | 19,205 | | 4,604 | | 13,692 | | 6,783 | |  |
| Hispanic | 151,238 | 57,997 | | 9,579 | | 16,036 | | 12,008 | | 15,557 | | 7,990 | |  |
| NHA | 141,675 | 57,806 | | 13,474 | | 17,868 | | 9,997 | | 10,520 | | 4,915 | |  |
| Other/Unknown | 16,203 | 5,018 | | 867 | | 1,559 | | 1,154 | | 1,772 | | 910 | |  |
| SEER Summary Stage at diagnosis of index (a) |  |  | |  | |  | |  | |  | |  | |  |
| Local | 703,570 | 359,776 | | 33,313 | | 65,739 | | 48,381 | | 18,873 | | 52,714 | |  |
| Regional | 382,571 | 176,411 | | 40,422 | | 64,159 | | 19,759 | | 12,117 | | 10,750 | |  |
| Distant | 271,461 | 27,225 | | 80,798 | | 30,146 | | 2,355 | | 80,381 | | 6,865 | |  |
| Unknown | 125,414 | 10,280 | | 9,959 | | 7,361 | | 1,344 | | 22,371 | | 2,902 | |  |
| Footnotes: |  |  |  | |  | |  | |  | |  | |  | |
| (a) SEER summary stage used for cases diagnosed 1998-2017, SEER historic summary staged used for cases diagnosed 1992-1997 | | | | | | | | | | |  | |  | |

| Supplementary Table 2. Demographics for subsequent cancer diagnosis | | |  | |  | |  | |  | |  | |  | |
| --- | --- | --- | --- | --- | --- | --- | --- | --- | --- | --- | --- | --- | --- | --- |
| Subsequent non-GYN cancer |  |  |  | |  | |  | |  | |  | |  | |
| Primary Site | All Non GYN Sites | Breast | | Lung and Bronchus | | Colon and Rectum | | Thyroid | | Hematopoietics | | Urinary System | |  |
| Total | 17,675 | 5,442 | | 2,464 | | 2,327 | | 636 | | 1,606 | | 1,282 | |  |
| Age of diagnosis |  |  | |  | |  | |  | |  | |  | |  |
| 18-49 | 1,184 | 321 | | 129 | | 141 | | 169 | | 97 | | 75 | |  |
| 50-59 | 2,812 | 914 | | 340 | | 349 | | 155 | | 225 | | 216 | |  |
| 60-69 | 5,015 | 1,735 | | 677 | | 595 | | 188 | | 415 | | 366 | |  |
| 70+ | 8,664 | 2,472 | | 1,318 | | 1,242 | | 124 | | 869 | | 625 | |  |
| Year of diagnosis |  |  | |  | |  | |  | |  | |  | |  |
| 1992-1999 | 1,822 | 576 | | 293 | | 274 | | 31 | | 155 | | 144 | |  |
| 2000-2009 | 6,551 | 1,932 | | 1,016 | | 974 | | 195 | | 569 | | 454 | |  |
| 2010-2018 | 9,302 | 2,934 | | 1,155 | | 1,079 | | 410 | | 882 | | 684 | |  |
| Race/Ethnicity |  |  | |  | |  | |  | |  | |  | |  |
| NHW | 13,050 | 4,088 | | 1,834 | | 1,679 | | 402 | | 1,203 | | 936 | |  |
| NHB | 1,323 | 346 | | 224 | | 205 | | 33 | | 124 | | 114 | |  |
| Hispanic | 1,717 | 473 | | 196 | | 218 | | 99 | | 154 | | 148 | |  |
| NHA | 1,471 | 512 | | 189 | | 210 | | 97 | | 113 | | 76 | |  |
| Other/Unknown | 114 | 23 | | 21 | | 15 | | * | | 12 | | * | |  |
| SEER Summary Stage |  |  | |  | |  | |  | |  | |  | |  |
| Local | 7,598 | 3,524 | | 530 | | 918 | | 411 | | 219 | | 850 | |  |
| Regional | 3,586 | 1,223 | | 512 | | 766 | | 135 | | 110 | | 152 | |  |
| Distant | 3,482 | 218 | | 1,121 | | 368 | | 25 | | 980 | | 132 | |  |
| Unknown | 3,009 | 477 | | 301 | | 275 | | 65 | | 297 | | 148 | |  |
| Subsequent GYN cancer |  |  |  | |  | |  | |  | |  | |  | |
| Primary Site | All GYN Sites | Cervix Uteri | | Corpus Uteri and Uterus, NOS | | Ovary | | Vulva/Vaginal | |  | |  | |  |
| Total | 14,670 | 859 | | 8,702 | | 3,628 | | 1,010 | |  | |  | |  |
| Age of diagnosis |  |  | |  | |  | |  | |  | |  | |  |
| 18-49 | 1,013 | 172 | | 444 | | 315 | | 53 | |  | |  | |  |
| 50-59 | 2,632 | 153 | | 1,619 | | 651 | | 121 | |  | |  | |  |
| 60-69 | 3,985 | 178 | | 2,596 | | 867 | | 217 | |  | |  | |  |
| 70+ | 7,040 | 356 | | 4,043 | | 1,795 | | 619 | |  | |  | |  |
| Year of diagnosis |  |  | |  | |  | |  | |  | |  | |  |
| 1992-1999 | 1,609 | 136 | | 920 | | 444 | | 88 | |  | |  | |  |
| 2000-2009 | 5,579 | 328 | | 3,255 | | 1,518 | | 377 | |  | |  | |  |
| 2010-2018 | 7,482 | 395 | | 4,527 | | 1,666 | | 545 | |  | |  | |  |
| Race/Ethnicity |  |  | |  | |  | |  | |  | |  | |  |
| NHW | 10,951 | 557 | | 6,448 | | 2,783 | | 801 | |  | |  | |  |
| NHB | 1,159 | 98 | | 729 | | 233 | | 68 | |  | |  | |  |
| Hispanic | 1,263 | 107 | | 721 | | 311 | | 87 | |  | |  | |  |
| NHA | 1,208 | 86 | | 755 | | 278 | | 51 | |  | |  | |  |
| Other/Unknown | 89 | 11 | | 49 | | 23 | | * | |  | |  | |  |
| SEER Summary Stage |  |  | |  | |  | |  | |  | |  | |  |
| Local | 6,680 | 332 | | 5,385 | | 395 | | 506 | |  | |  | |  |
| Regional | 2,780 | 295 | | 1,650 | | 589 | | 188 | |  | |  | |  |
| Distant | 3,228 | 112 | | 703 | | 2,221 | | 32 | |  | |  | |  |
| Unknown | 1,982 | 120 | | 964 | | 423 | | 284 | |  | |  | |  |
